# Supplementary material for: NRAS destines tumor cells to the lungs
Source: EMBO Mol Med. 2017 Mar 24;9(5):672–86. doi: 10.15252/emmm.201606978 (PMC5697015; doi:10.15252/emmm.201606978)
Supplement: Supplementary file 1 — Appendix [file EMMM-9-672-s001.pdf]

APPENDIX

Research Article

NRAS destines tumor cells to the lungs

Anastasios D. Giannou, Antonia Marazioti, Nikolaos I. Kanellakis, Ioanna Giopanou, Ioannis Lilis, Dimitra E. Zazara, Giannoula Ntaliarda, Danai Kati, Vasileios Armenis, Georgia A. Giotopoulou, Anthi C. Krontira, Marina Lianou, Theodora Agalioti, Malamati Vreka, Maria Papageorgopoulou, Sotirios Fouzas, Dimitrios Kardamakis, Ioannis Psallidas, Magda Spella, and Georgios T. Stathopoulos.

TABLE OF CONTENTS

	Pages
**APPENDIX SUPPLEMENTARY TABLES**	
**Appendix Table S1 -** Top 20 of 107 transcripts overrepresented more than two-fold in LLC cells stably expressing anti-*Nras* shRNA compared with control cells expressing random shRNA	2
**Appendix Table S2 -** Top 20 of 154 transcripts underrepresented more than two-fold in LLC cells stably expressing anti-*Nras* shRNA compared with control cells expressing random shRNA	3
**Appendix Table S3 -** Top 20 of 1110 transcripts overrepresented more than two-fold in AE17 cells stably expressing anti-*Nras* shRNA compared with control cells expressing random shRNA	4
**Appendix Table S4 -** Top 20 of 901 transcripts underrepresented more than two-fold in AE17 cells stably expressing anti-*Nras* shRNA compared with control cells expressing random shRNA	5
**Appendix Table S5 -** PCR primers used for these studies	6-7
**Appendix Table S6 -** Antibodies used for these studies	8

Appendix Table S1 - Top 20 of 107 transcripts overrepresented more than two-fold in LLC cells stably expressing anti-*Nras* shRNA compared with control cells expressing random shRNA

Gene symbol	Gene name	ΔGE[a]
Itgb7	integrin beta 7	+4.57
Cd68	CD68 antigen	+3.85
Rnu3a	U3A small nuclear RNA	+3.72
Tuba1b	tubulin, alpha 1B	+3.21
Acta2	actin, alpha 2, smooth muscle, aorta	+2.92
Pfkl	phosphofructokinase, liver, B-type	+2.88
Nptx1	neuronal pentraxin 1	+2.87
Ndrg1	N-myc downstream regulated gene 1	+2.80
Hist1h2bk	histone cluster 1, H2bk	+2.66
Acy3	aspartoacylase (aminoacylase) 3	+2.65
Glt8d2	glycosyltransferase 8 domain containing 2	+2.58
Snora73a	small nucleolar RNA, H/ACA box 73a	+2.57
Snora44	small nucleolar RNA, H/ACA box 44	+2.53
Tppp3	tubulin polymerization-promoting protein family member 3	+2.53
Krt18	keratin 18	+2.53
Pgam1	phosphoglycerate mutase 1	+2.48
Fam83d	family with sequence similarity 83, member D	+2.47
Hist1h2bn|l|j|f	histone cluster 1, H2bn | l | j | f	+2.46
Tigit	T cell immunoreceptor with Ig and ITIM domains	+2.41
Lpin1	lipin 1	+2.41

[a] *Δ*GE, difference in gene expression between LLC cells stably expressing anti-*Nras* shRNA compared with control cells expressing random shRNA.

Appendix Table S2 - Top 20 of 154 transcripts underrepresented more than two-fold in LLC cells stably expressing anti-*Nras* shRNA compared with control cells expressing random shRNA

Gene symbol	Gene name	ΔGE[a]
Cxcl5	chemokine (C-X-C motif) ligand 5	-16.30
Ranbp3l	RAN binding protein 3-like	-7.71
Phxr4	per-hexamer repeat gene 4	-4.55
Mir763	microRNA 763	-3.91
5730469M10Rik	RIKEN cDNA 5730469M10 gene	-3.81
Slc7a11	solute carrier family 7, member 11	-3.62
Gm20199	predicted gene, 20199	-3.60
Ccl2	chemokine (C-C motif) ligand 2	-3.59
Ccl7	chemokine (C-C motif) ligand 7	-3.55
Saa3	serum amyloid A 3	-3.43
Gm7265	predicted gene 7265	-3.39
C030013D06Rik	RIKEN cDNA C030013D06 gene	-3.35
Fgf7	fibroblast growth factor 7	-3.32
Gm4787	predicted gene 4787	-3.29
Mir3094	microRNA 3094	-3.29
Zfp932	zinc finger protein 932	-3.27
9930017N22Rik|LOC100503407	RIKEN cDNA 9930017N22 gene | uncharacterized LOC100503407	-3.21
Gm19270	predicted gene, 19270	-3.07
Fabp4	fatty acid binding protein 4, adipocyte	-3.01
Igfbp7	insulin-like growth factor binding protein 7	-2.96

[a] *Δ*GE, difference in gene expression between LLC cells stably expressing anti-*Nras* shRNA compared with control cells expressing random shRNA. *Cxcl5* was studied further.

Appendix Table S3 - Top 20 of 1110 transcripts overrepresented more than two-fold in AE17 cells stably expressing anti-*Nras* shRNA compared with control cells expressing random shRNA

Gene symbol	Gene name	ΔGE[a]
2310002L13Rik	RIKEN cDNA 2310002L13 gene	+17.92
Lox	lysyl oxidase	+17.47
Hist1h2bk	histone cluster 1, H2bk	+16.50
Ccl5	chemokine (C-C motif) ligand 5	+15.32
Pcsk9	proprotein convertase subtilisin/kexin type 9	+15.09
Cdh2	cadherin 2	+14.65
Lipg	lipase, endothelial	+14.27
Btg3	B cell translocation gene 3	+13.48
Efna5	ephrin A5	+13.06
Manea	mannosidase, endo-alpha	+12.50
Zfhx4	zinc finger homeodomain 4	+12.41
F11r	F11 receptor	+12.08
Nfib	nuclear factor I/B	+11.65
Sdc3	syndecan 3	+11.50
Gpr126	G protein-coupled receptor 126	+11.49
Dnmbp	dynamin binding protein	+11.14
Colec12	collectin sub-family member 12	+11.12
Fn1	fibronectin 1	+10.95
Tnc	tenascin C	+10.81
Nrn1	neuritin 1	+10.61

[a] *Δ*GE, difference in gene expression between AE17 cells stably expressing anti-*Nras* shRNA compared with control cells expressing random shRNA.

Appendix Table S4 - Top 20 of 901 transcripts underrepresented more than two-fold in AE17 cells stably expressing anti-*Nras* shRNA compared with control cells expressing random shRNA

Gene symbol	Gene name	ΔGE[a]
Ppbp	pro-platelet basic protein	-38.83
C1qtnf3	C1q and tumor necrosis factor related protein 3	-18.08
Ptn	pleiotrophin	-13.59
A430107O13Rik	RIKEN cDNA A430107O13 gene	-12.36
Cdk15	cyclin-dependent kinase 15	-12.26
Timp4	tissue inhibitor of metalloproteinase 4	-12.01
Bmp5	bone morphogenetic protein 5	-11.84
Adm	adrenomedullin	-10.72
Prph	peripherin	-10.58
Wfdc15a	WAP four-disulfide core domain 15A	-10.55
Gm364	predicted gene 364	-10.49
Gm5077	predicted gene 5077	-10.17
Rhox2g	reproductive homeobox 2G	-9.70
Ogn	osteoglycin	-9.29
Nxf3	nuclear RNA export factor 3	-9.19
Rcn3	reticulocalbin 3, EF-hand calcium binding domain	-9.13
Dock10	dedicator of cytokinesis 10	-9.11
Dkk2	dickkopf homolog 2 (Xenopus laevis)	-9.07
Pla2g4a	phospholipase A2, group IVA	-8.89
Actc1	actin, alpha, cardiac muscle 1	-8.83

[a] ΔGE, difference in gene expression between AE17 cells stably expressing anti-*Nras* shRNA compared with control cells expressing random shRNA. *Ppbp* was studied further.

Appendix Table S5 - PCR primers used for these studies

Method[a]	Primer	Sequence	Amplicon (bp)
Seq	KrasF	CCATTTCGGACCCGGAG	904
	KrasR	CTTTAGTCTCTTCCACAGGCA	
	NrasF	GCGCCTAGTGATTACGTAGC	905
	NrasR	TGAAGAGGTCTCAGGTTAGATGG	
	Hras1F	CGGAGCTCATGCGCGG	881, 734
	Hras1R	TCCGGCACCTCCATGTCCT	
	Hras1F2	ACACAGCAGGTCAAGAAGAG	577, 430
	Hras1R	TCCGGCACCTCCATGTCCT	
	mEgfrF1	GCCTGATAACTGGACTGACCT	F1/R1 1673; F2R2
	mEgfrR1	AGAATCAACTCTCGGAACTTTGG	2261; F2/R 2073;
	mEgfrF2	CTCCTCTTCTTCCCGCACTG	1S1/R1 1174;
	mEgfrR2	AGAATCTGAGACCTCTGGCTG	1S2/R1 672;
	mEgfrR	GCATAGGTGGCAGACATTATTGG	1S1/R2 1759
	mEgfr1S1	ACAACTGCATCCAGTGTGCC	1S1/R 1571;
	mEgfr1S2	GGCCATCAAGGAGTTAAGAG	2S2/R2 1256;
	mEgfr2S1	AGAGAATCCCTTTGGAGAACC	2S2/R 1068;
	mEgfr2S2	CCACCACTCATGCTGTACAACC	2S3/R2 731; 2S3/R
	mEgfr2S3	GTCGTTGGCCTGAACATCAC	543
	Pik3caF1	ATTCTGACTCCATAAGGCGG	1493
	Pik3caR1	GAACCAATCAAACTCCAACTC	
	Pik3caF2	GCTGAACCCTATTGGTGTTACTG	1958
	Pik3caR2	GCTCAAGTCCTAATGTTGTTCCT	
	BrafF	CGCTGTCTTCGGAAATACCA	1551
	BrafR	AATTCTTTCCATCATGCCTGACC	
	BrafF1	GGCGGGTTCCAGAGGTG	2066
	BrafR1	CACTCCACCGAGATTTCACTG	
	Trp53F	GTAGCTTCAGTTCATTGGGA	1354, 1450
	Trp53F	GAAGTCATAAGACAGCAAGGA	
	Stk11F	GGGACGAGGACAAAGAGTGG	1183, 1374
	Stk11R	CTGCCCAGAGATCCTGATGAC	
qPCR	NrasF	CCACCATAGAGGATTCTTACCGA	149
	NrasR	GGCAAATACACAGAGGAACCC	
	Cxcl5F	TTGGGCAGTGACAAAAAGAA	100
	Cxcl5R	TTCACTGGGGTCAGAGTCCT	
	PpbpF	GTACAGGCCAGGAGTTCACT	106
	PpbpR	GACGATTCTCTTGACGCCAG	
	Cxcr1F	TCTACGTGCTGAACCTAGCC	117
	Cxcr1R	GTGAGACCATCTTGCACAGG	
	Cxcr2F	TTCTGCTACGGGTTCACACT	128
	Cxcr2R	AGAACCAGGTTGTAGGGCAG	
	GusbF	TTACTTTAAGACGCTGATCACC	165
	GusbR	ACCTCCAAATGCCCATAGTC	

GapdhF	TGTGTCCGTCGTGGATCTGA	150
GapdhR	TTGCTGTTGAAGTCGCAGGAG	
HprtF	AGTCCCAGCGTCGTGATTAG	88
HprtR	TTTCCAAATCCTCGGCATAATGA	
NRASF	TCCAGCTAATCCAGAACCACT	148
NRASR	TGGTCTCTCATGGCACTGTA	
CXCL6F	CGAACCCTCTCTTGACCACT	149
CXCL6R	CACAGCAGAGACAGGACCAG	
CXCL7F	CGAAAGGCAAAGAGGAAAGTC	120
CXCL7R	CTTTCCCGATCACTTCCAAA	
CXCL8F	GCAGAGGGTTGTGGAGAAGTT	150
CXCL8R	ACCCTACAACAGACCCACAC	
GUSBF	CTACTTGAAGATGGTGATCGCTC	118
GUSBR	ACAGATCACATCCACATACGG	

[a]Application: Seq, sequencing; qPCR, quantitative (real-time) PCR.

Appendix Table S6 - Antibodies used for these studies

Method[a]	Target	Provider[b]	Catalog #	Dilution	Conjugate[c]
IF	CXCR1	Abcam	Ab10400	1:20	-
	CXCR2		Ab14935	1:100	-
	GFP	Santa Cruz	sc-8334	1:200	-
	CD34	eBioscience	11-0341	1:200	FITC
IH	PCNA	Abcam	ab2426	1:2000	-
FC	CD11b	eBioscience	12-0112	0.1 µg / 10^6 cells	PE
	Ly6C		45-5932-82		PerCP- Cy5.5
	Gr1		25-5931-82		PE-Cy7
WIB	NRAS	Santa Cruz	sc-31	1:500	-
	β-actin		sc-47778	1:500	-
	Goat anti-rabbit & anti-mouse IgG	Southern Biotech	4030-05 & 1030-05	1:8000	HRP
IF	Donkey anti-rabbit & anti-mouse IgG	Molecular Probes	A21206	1:1000	AF488
			A21202		AF488
			A10042		AF568
			A10037		AF568
			A31573		AF647
			A31571		AF647

a Application: IF, immunofluorescence; WIB, Western immunoblotting; IH, immunohistochemistry; FC, flow cytometry.

b Providers*:* Abcam, Cambridge, UK; eBioscience, San Diego, CA; Santa Cruz Biotechnology, Dallas, TX; Southern Biotech, Birmingham, AL; Molecular Probes, Eugene, OR.

c Conjugates*:* FITC, fluorescein isothiocyanate; PE, phycoerythrin; PerCP, peridinin-chlorophyll protein; HRP, horse radish peroxidase; AF, Alexa Fluor.
