# Supplementary material for: NRAS destines tumor cells to the lungs
Source: EMBO Mol Med. 2017 Mar 24;9(5):672–86. doi: 10.15252/emmm.201606978 (PMC5697015; doi:10.15252/emmm.201606978)
Supplement: Supplementary file 2 — Expanded View Figures PDF [file EMMM-9-672-s002.pdf]

Expanded View Figures

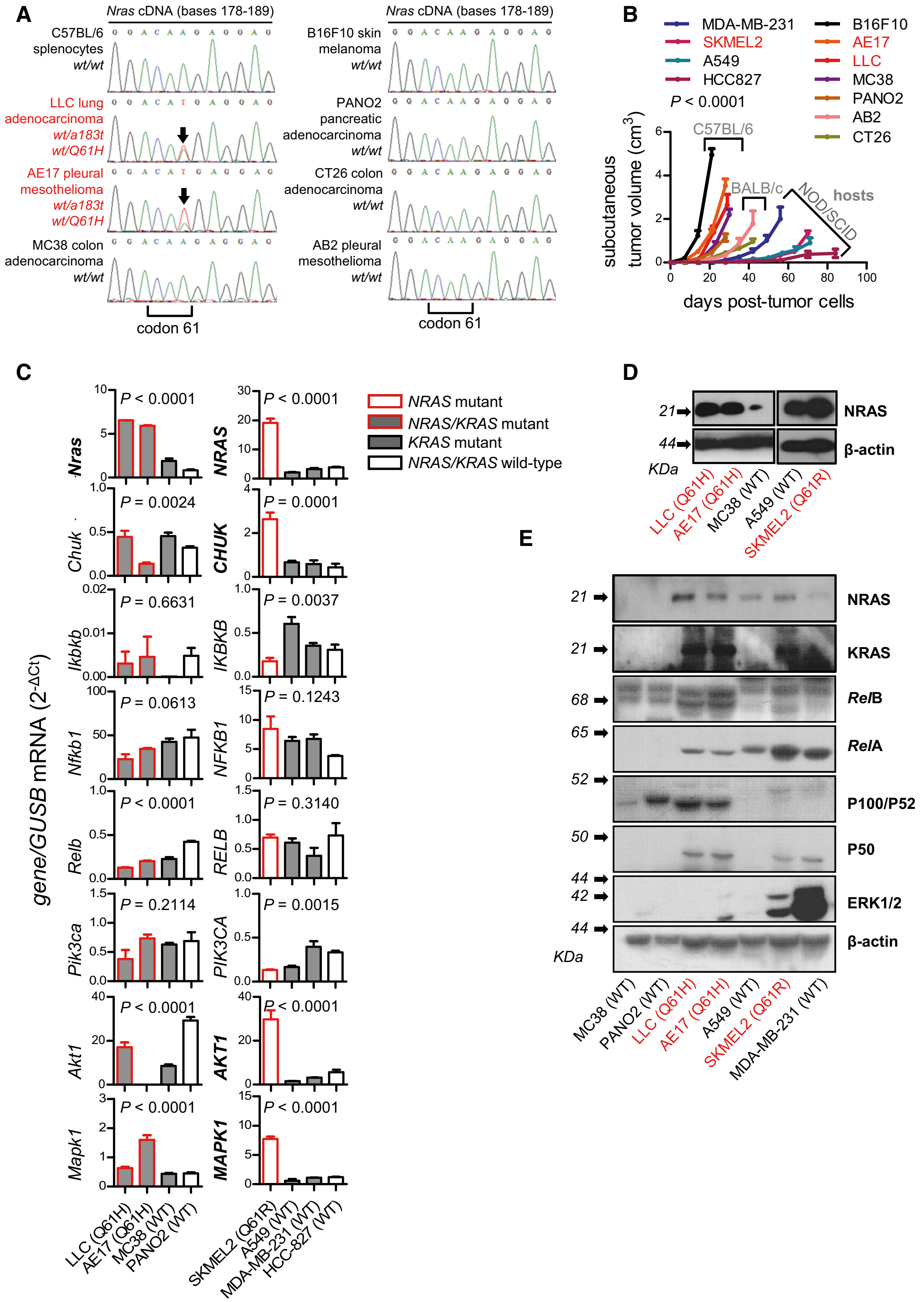

Figure EV1.

 © 2017 The Authors

Figure EV1. *Nras* mutations of murine cancer cell lines.

A Representative Sanger sequencing traces of *Nras* codons 60–63 of some mouse cancer cell lines employed in these studies showing *Nras* mutations (red font, black arrows). Shown is one representative of three traces.

B Weekly monitored primary tumor volume of C57BL/6, BALB/c, and NOD/SCID host mice after s.c. delivery of 0.5 × 10^6 mouse or 10^6 human tumor cells (*n* for each group is given in Fig 1E, table).

C–E mRNA and protein of mouse and human tumor cell lines harboring wild-type (WT) and mutant *NRAS* and *KRAS* alleles were examined by qPCR (C, *n* = 3) and immunoblotting (D, E; shown are one representative of three experiments).

Data information: Cell lines are described in the text. All data are presented as mean ± SEM. *P*: probability by two-way ANOVA (B) and one-way ANOVA (C). (B, D, E): *NRAS*-mutant cell lines are in red font. (C): Genes overexpressed specifically by *NRAS*-mutant cell lines are in bold font.

Source data are available online for this figure.

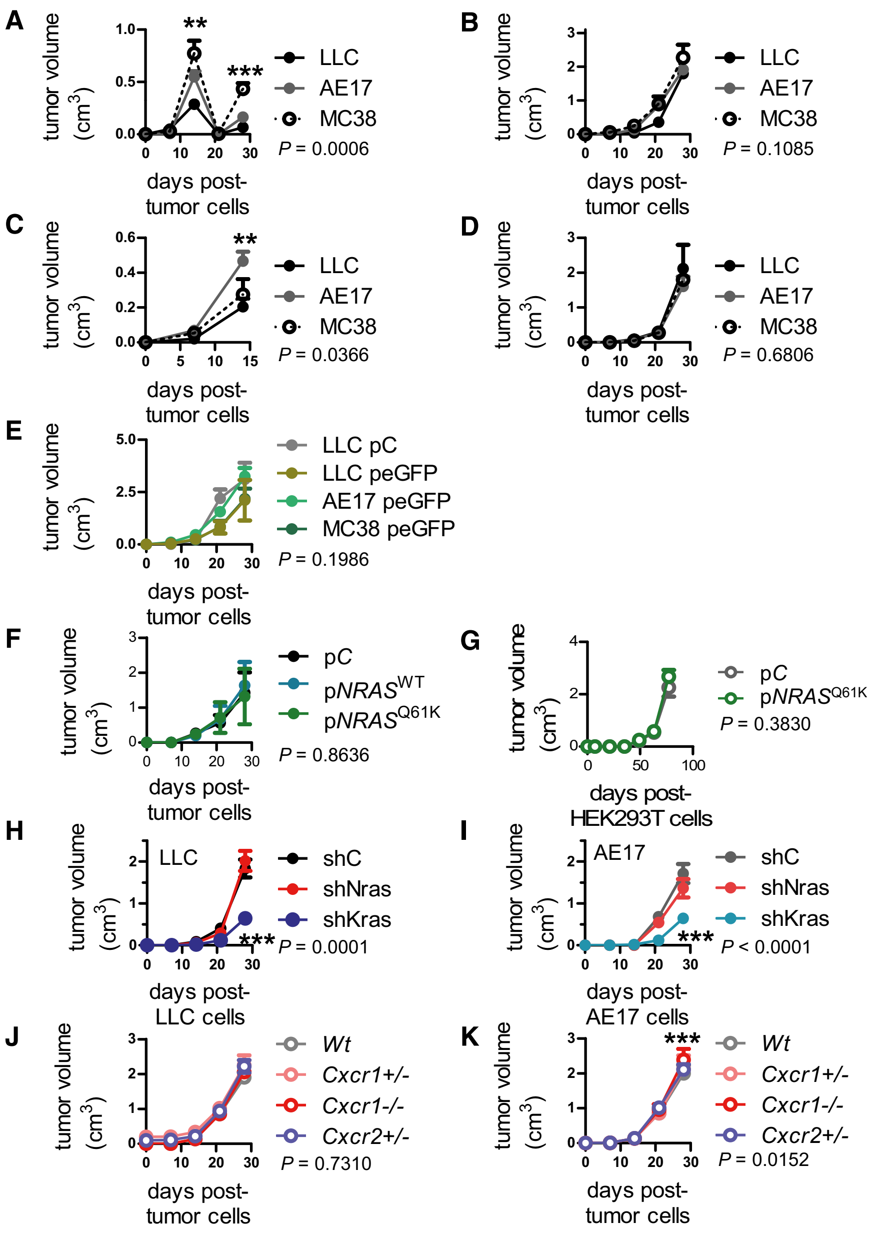

Figure EV2. Primary tumor volume of experiments from Figs 2–4 and 7.

A Primary tumor volume of experiment from Fig 2A (*n* = 3 mice/cell line).

B Primary tumor volume of experiment from Fig 2B (*n* = 5 mice/cell line).

C Primary tumor volume of experiment from Fig 2C (*n* = 5 mice/cell line).

D Primary tumor volume of experiment from Fig 2D (*n* = 4 mice/cell line).

E Primary tumor volume of experiment from Fig 3A (*n* = 4 mice/cell line).

F Primary tumor volume of experiment from Fig 3D (*n* = 8 mice/cell line).

G Primary tumor volume of experiment from Fig 3E (*n* = 7–8/cell line).

H, I Primary tumor volume of experiments from Fig 4C (*n* is given in Fig 4C, tables).

J, K Primary tumor volume of experiments from Fig 7A (*n* is given in Fig 7A, tables).

Data information: Cell lines are described in the text. All data are presented as mean ± SEM. *P*: probability by two-way ANOVA. ** and ***: $P < 0.01$ and $P < 0.001$ compared to controls at the time point indicated by Bonferroni post-test.

© 2017 The Authors

A

Mus musculus *Nras* (GenBank Accession ID: NM_010937.2)

1 gggactgggg cgccttgggc gcctagtgat tacgtagcgg gtggggccgg aagtgccgct
61 ccctggcggg ggctgttcat ggcggtttcg gggtctccaa cagcttctca ggttgaagtc
121 caaaagcctc ccgaggcggg gtctgcggag tttgaggttt ttgctggtgt gaaatgactg
181 agtacaaact ggtggtggtt ggagcaggtg gtgttgggaa aagcgccttg acgatccagc
241 taatccagaa ccactttgtg gatgaatatg atcccaccat agaggattct taccgaaagc
301 aagtggtgat tgatggtgag acctgcctgc tggacatact ggacacagct ggacaagagg
361 agtacagtgc catgagagac cagtacatga ggacaggcga agggttcctc tgtgtatttg
421 ccatcaataa tagcaaatca tttgcagata ttaacctcta cagggagcaa attaagcgtg
481 tgaaagattc tgatgatgtc cccatggtgc tggtaggcaa caagtgtgac ttgccaa**caa**
541 **ggacagttga cacaaa**gcaa gcccacgaac tggccaagag ttacggaatt ccattcattg
601 agacctcagc caagacccga cagggtgtgg aggatgcctt ttacacactg gtaagggaga
661 tacgccagta ccgaatgaaa aagctcaaca gcagtgacga tggcactcaa ggttgtatgg
721 ggctgccctg tgtgctgatg tagtaagaca ctttgaaagt tctgtcatca gaaaagagcc
781 actttgaagc tgcactgatg ccctggttct gacatccctg gaggagacct gttcctgctg
...
1321 taaatccaac attcacaaca aagtgccttt ttcctaaaag tgctctgtag gctccattac
1381 agtttgtaat t**ggaatagat gtgtcaagaa** ccattgtata ggaaagtgac tctgagccat
1441 ctacctttga gggaaaggtg tatgtacctg atggcagatg ctttgtgtat gcacatgaag
...
2701 tacaggcaca ttccaccaca cacacctgac tgagctcgtt cctaatgagt tttcattaag
2761 caaattc**ccc atcaccttga aactaa**tcag aagggggaag aaacatttgc tatgctcctg
2821 agtgctaaca ctgggatcat tcacatgggg tttgcattcc taggcaaact aaactgctgc
...

B

Mus musculus *Kras* (GenBank Accession ID: NM_021284.6)

1 aggcggcggc cgcggcggct gaggcggcag cgctgtggcg gcggctgaga cggcagggga
61 aggcggcggc ggctcggccc ggagtcccgc tcccgcgcca tttcggaccc ggagcgagcg
121 cggcgcgggc ctgaaggcgg cggcgggagc ctgaggcgcg gcggctccgc ggcgcggaga
181 gaggcctgct gaaaatgact gagtataaac ttgtggtggt tggagctggt ggcgtaggca
241 agagcgcctt gacgatacag ctaattcaga atcactttgt ggatgagtat gaccctacga
301 tagaggactc **ctacaggaaa caagtagta**a ttgatggaga aacctgtctc ttggatattc
361 tcgacacagc aggtcaagag gagtacagtg caatgaggga ccagtacatg agaactgggg
421 agggctttct ttgtgtattt gccataaata atactaaatc atttgaagat attcaccatt
481 atagagaaca aattaaaaga gtaaaggact ctgaagatgt gcctatggtc ctggtaggga
541 ataagtgtga tttgccttct a**gaacagtag acacgaaaca** ggctcaggag ttagcaagga
601 gttacgggat tccgttcatt gagacctcag caaagacaag acagggtgtt gacgatgcct
661 tctatacatt agtccgagaa attcgaaaac ataaagaaaa gatgagcaaa gatgggaaga
721 agaagaagaa gaagtcaagg acaaggtgta cagttatgtg aatactttgt actctttctt
...
1321 tatttttttct agaaaagggg aaaaatggaa aaaaaaaggc aatggaaaat gttgaaat**cc**
1381 **attcagtttc catgtta**gct aaattactgt aagattccta taatagcttt tcctggtaag
1441 gcagacccag tatgaaatag taataaccat ttgggctata tttacatgct actaaatttt
...

Figure EV3. Target specificity of shRNAs against murine *Nras* and *Kras* transcripts.

Partial *Mus musculus Nras* (A) and *Kras* (B) transcripts with start (green boxes) and stop (red boxes) codons, showing the target sequences of the anti-*Nras* (red font) and anti-*Kras* (blue font) shRNA pools employed in the present study. Note that there is no sequence homology between *Nras*- and *Kras*-specific target sequences and that no *Nras*-specific target sequence is present in the *Kras* transcript and *vice versa*.

 © 2017 The Authors

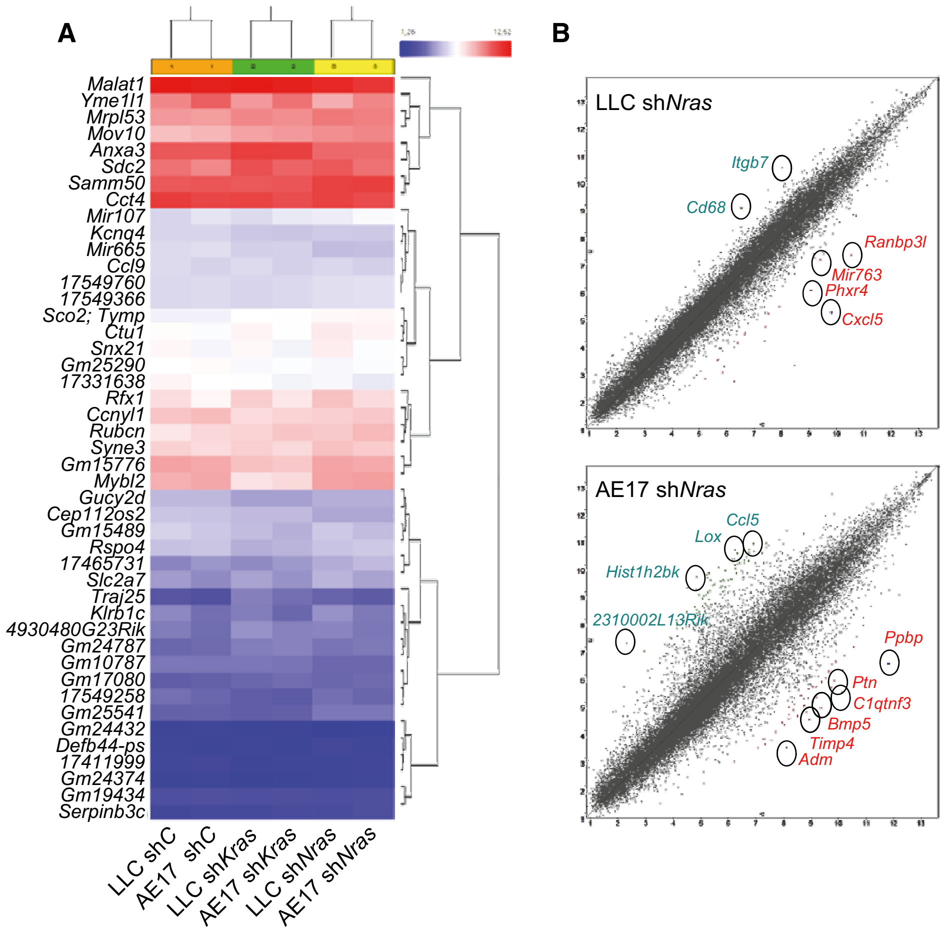

Figure EV4. Microarray comparing differential gene expression (ΔGE) of *Nras*^{Q61H}-mutant mouse tumor cells (LLC and AE17) expressing control (shC), anti-Kras (shKras), or anti-Nras (shNras) shRNAs.

A Hierarchical clustering using paired ANOVA cutoff probability value of $P < 0.001$ identifies 45 differentially expressed genes and clusters cells by silenced gene and not by cell line.

B Volcano plots of changes in gene expression by cell line showing the top altered genes. Transcripts downregulated by sh*Nras* are shown in red font and transcripts upregulated in green font. The distance from diagonal lines represent probability value.

© 2017 The Authors
