# Supplementary material for: NRAS destines tumor cells to the lungs
Source: EMBO Mol Med. 2017 Mar 24;9(5):672–86. doi: 10.15252/emmm.201606978 (PMC5697015; doi:10.15252/emmm.201606978)

Source Data. Immunoblots from Figure EV1D
Dashed outlines indicate blot areas shown in main Figure.

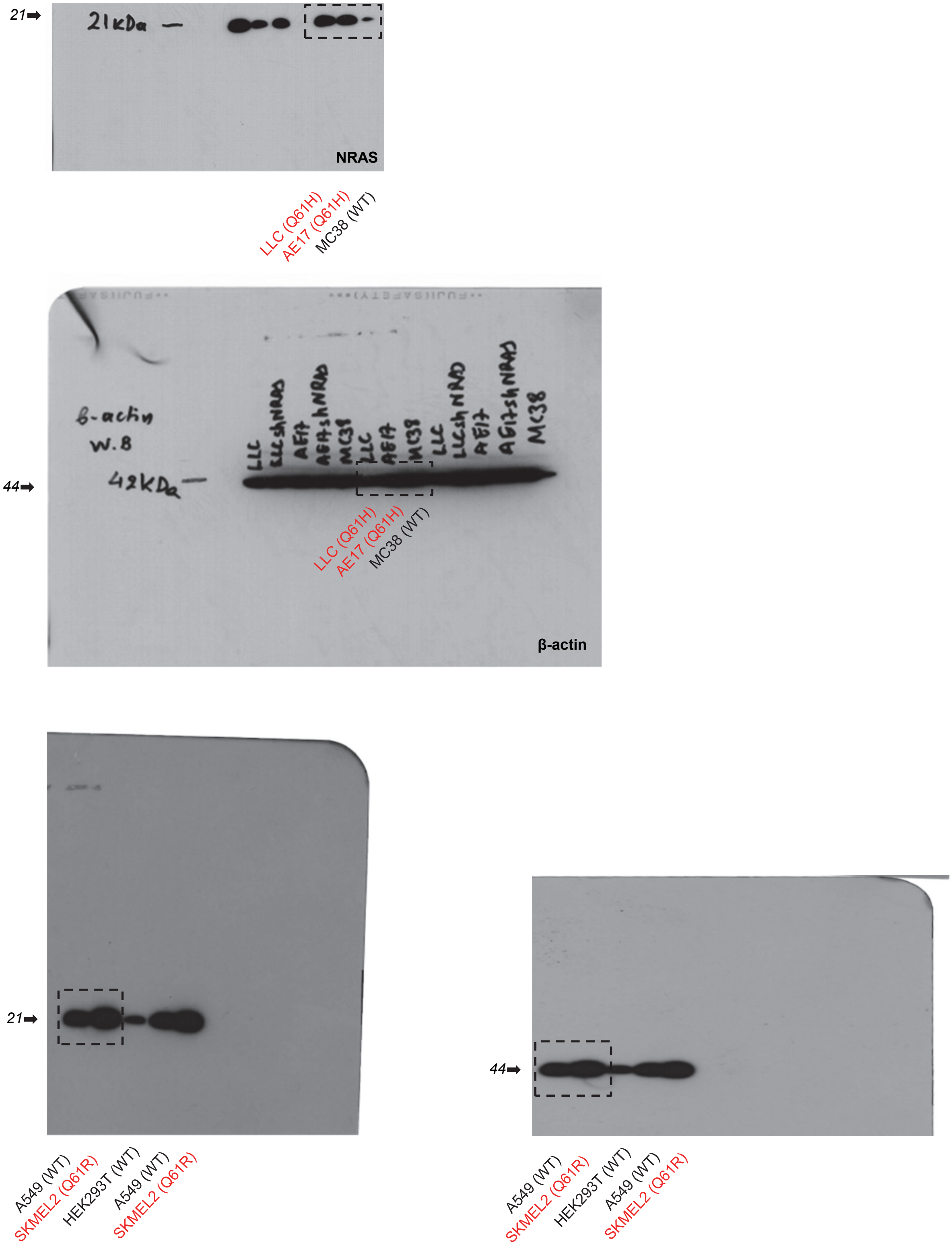

Source Data. Immunoblots from Figure EV1E
Dashed outlines indicate blot areas shown in main Figure.

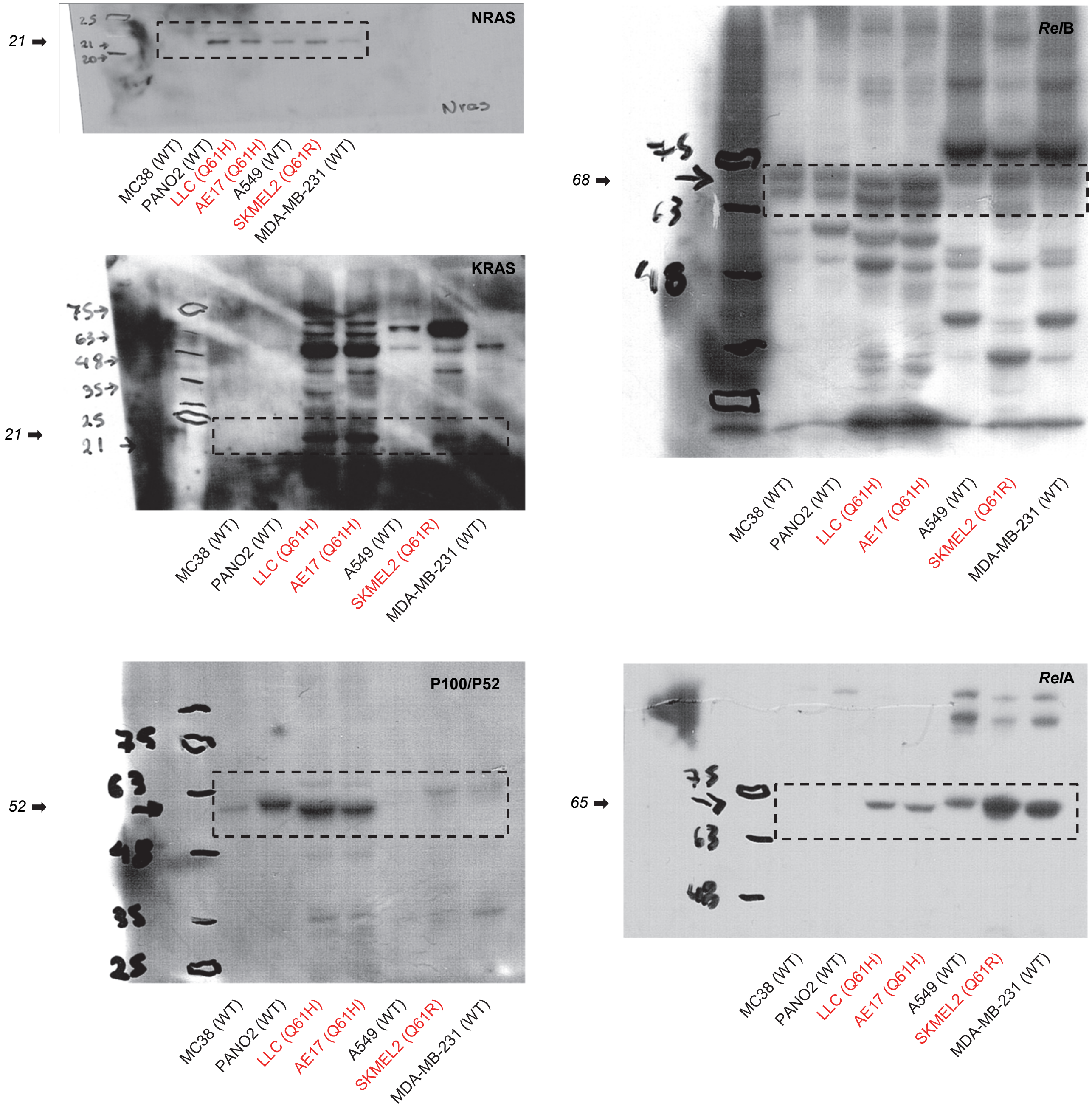

Source Data. Immunoblots from Figure EV1E
Dashed outlines indicate blot areas shown in main Figure.

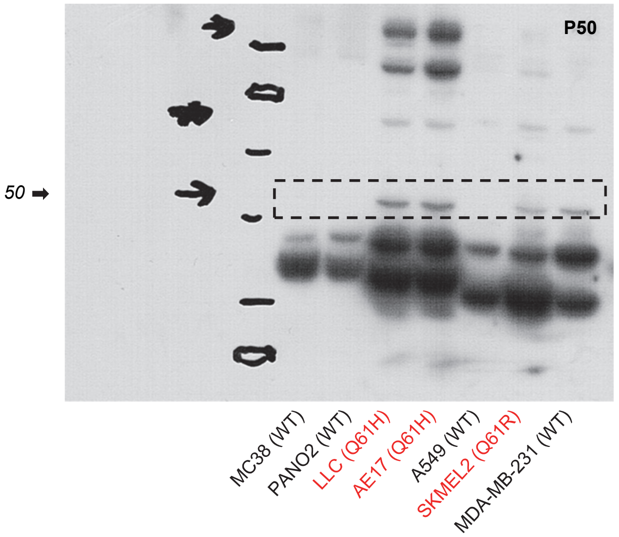

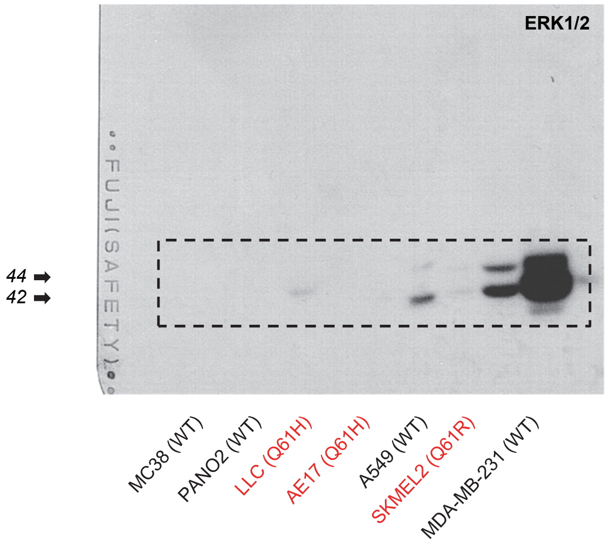

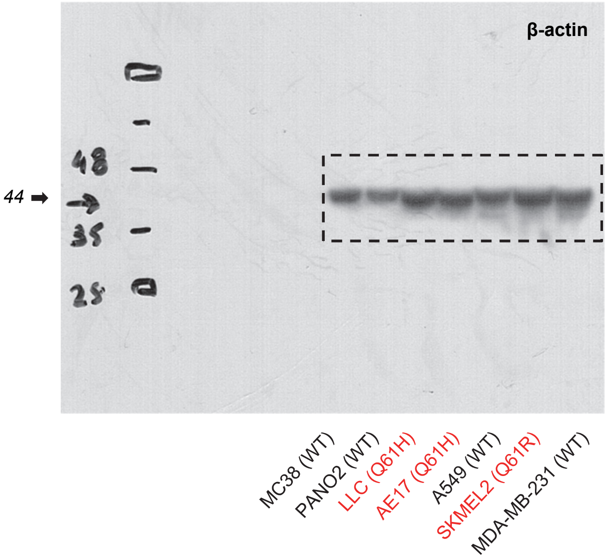

Supplement: Supplementary file 3 — Source Data for Expanded View [file EMMM-9-672-s003.zip › EMM201606978_V3_EVFig1_source_data_blots.pdf]
