# Supplementary material for: NRAS destines tumor cells to the lungs
Source: EMBO Mol Med. 2017 Mar 24;9(5):672–86. doi: 10.15252/emmm.201606978 (PMC5697015; doi:10.15252/emmm.201606978)
Supplement: Supplementary file 6 — Source Data for Figure 3 [file EMMM-9-672-s005.pdf]

Source Data. Immunoblots from Figure 3B
Dashed outlines indicate blot areas shown in main Figure.

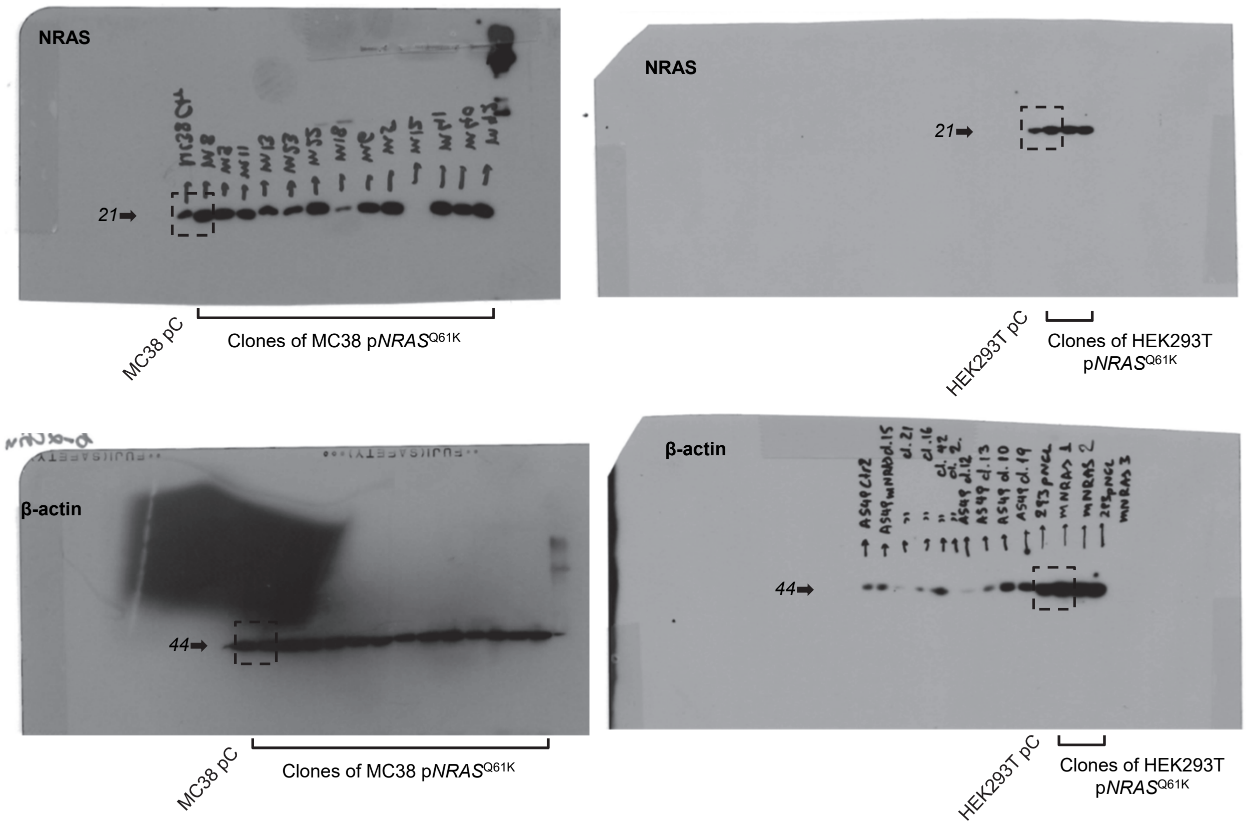

Source Data. Immunoblots from Figure 3D
Dashed outlines indicate blot areas shown in main Figure.

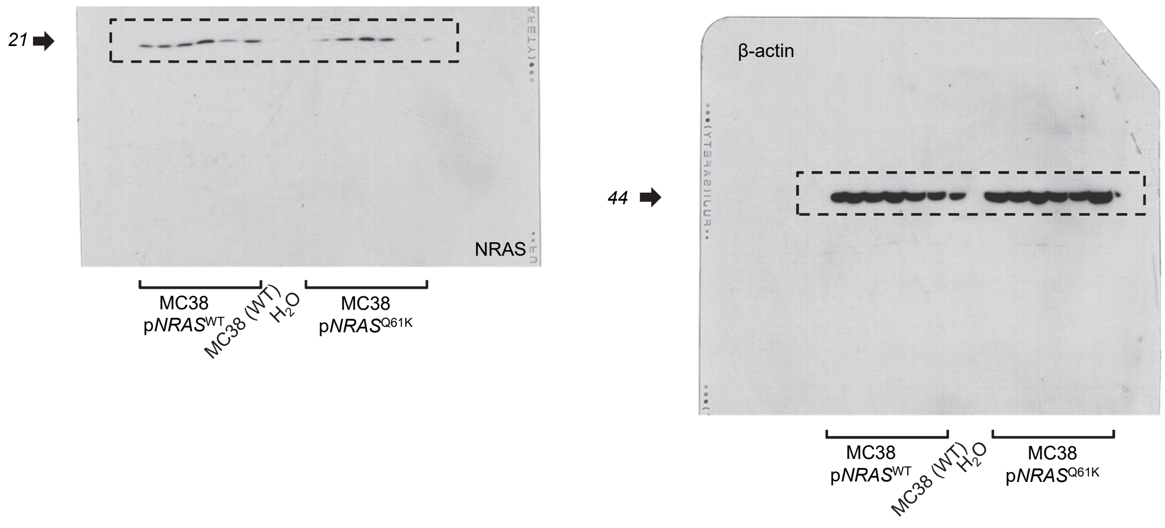

Fig 3D **Number of macrometastases of C57BL/6 mice at four weeks after s.c. delivery of 10^6 MC38 cells expressing pC, pNRASWT, and pNRASQ61K plasmids**

pC	pNRASWT	pNRASQ61K
0	1	5
0	1	0
0	5	2
0	2	2
0	3	3
0	0	0
0	3	3
0	0	2

Fig 3E **Fraction of the lungs affected by metastases of NOD/SCID mice at eleven weeks after s.c. or i.v. delivery of 3 x 10^6 HEK293T cells expressing pC and pNRASQ61K plasmids**

s.c.

pC	pNRASQ61K
0.013	0.065
0.006	0.064
0.007	0.213
0.034	0.081
0.011	0.152
0.056	0.052
0.008	0.056
	0.055

s.c.

pC	pNRASQ61K
0.022	0.101
0.023	0.196
0.021	0.274
0.043	0.076
0.025	0.098
