# Supplementary material for: NRAS destines tumor cells to the lungs
Source: EMBO Mol Med. 2017 Mar 24;9(5):672–86. doi: 10.15252/emmm.201606978 (PMC5697015; doi:10.15252/emmm.201606978)
Supplement: Supplementary file 7 — Source Data for Figure 4 [file EMMM-9-672-s006.pdf]

Source Data. Immunoblots from Figure 4

Dashed outlines indicate blot areas shown in main Figure.

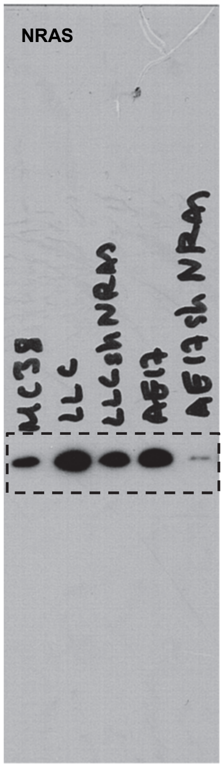

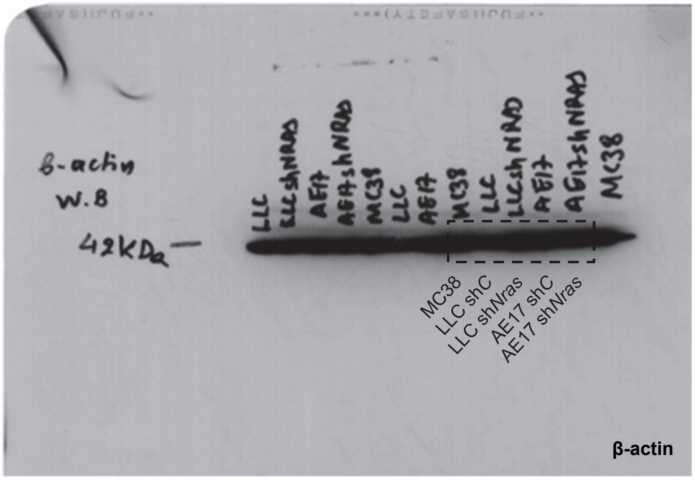

Fig 4C **Number of macrometastases of C57BL/6 mice at four weeks after s.c. delivery of 10^6 LLC or AE17 cells expressing shC, shNras, or shKras plasmids**

LLC

shC	shNras	shKras
7	0	0
5	0	1
8	0	0
5	3	0
3	1	1
1	0	0
3	1	0
3	1	2
19	1	
9	0	
8		
7		
3		
6		
5		
10		
4		
5		

AE17

shC	shNras	shKras
2	0	0
5	0	0
6	0	0
2	1	2
5	2	2
6	0	0
4	0	1
6	0	0
3		
4		
7		
1		
6		
6		
3		
5		

Fig 4D **Number of macrometastases of C57BL/6 mice at two weeks after i.v. delivery of 0.25 x 10^6 LLC or AE17 cells expressing shC or shNras, or MC38 cells**

LLC		AE17		MC38
shC	shNras	shC	shNras	
37	0	98	0	15
29	0	48	0	17
63	0	57	0	11
53	4	62	0	3
56	1	31	0	21
21	4	28	1	10
31	8			9
43	4			
