# Supplementary material for: NRAS destines tumor cells to the lungs
Source: EMBO Mol Med. 2017 Mar 24;9(5):672–86. doi: 10.15252/emmm.201606978 (PMC5697015; doi:10.15252/emmm.201606978)
Supplement: Supplementary file 8 — Source Data for Figure 7 [file EMMM-9-672-s007.pdf]

Fig 7A **Number of macrometastases of C57BL/6 wild-type (Wt) and Cxcr1/2 knockout mice at four weeks after s.c. delivery of 10^6 LLC or AE17 cells**

LLC

C57BL/6 (Wt	Cxcr1+/-	Cxcr1-/-	Cxcr2+/-
5	3	1	3
8	2	2	3
4	1	1	2
14	2	0	0
11	5	5	2
7	3	1	1
3	1	0	1
10	2	1	0
11	1	2	3
5	0	3	1
7	0		0
6			

AE17

C57BL/6 (Wt	Cxcr1+/-	Cxcr1-/-	Cxcr2+/-
16	11	0	1
0	10	2	0
46	3	2	2
6	1	0	1
34	1	0	1
4	2	0	3
18	0	2	0
24	2	1	0
14	1	3	3
1	1	1	2
3	2	1	0

Fig 7B **Number of macrometastases of C57BL/6 wild-type (Wt) and Cxcr1/2 knockout mice at two weeks after s.c. delivery of 0.25 x 10^6 LLC or AE17 cells**

LLC

C57BL/6 (Wt	Cxcr1-/-	Cxcr2+/-
26	3	1
33	0	2
29	2	1
11	1	2
12	3	3
	1	3
	1	1

AE17

C57BL/6 (Wt	Cxcr1-/-	Cxcr2+/-
163	63	21
87	39	32
134	61	4
182	42	30
76	12	7
102		31
78		45
89		71
142		42

Fig 7C **Number of macrometastases of irradiated C57BL/6 wild-type (Wt) and Cxcr1 knockout recipients after BMT from Wt, Cxcr1, or Cxcr2 knockout donors at four weeks after s.c. delivery of 10^6 LLC or AE17 cells**

		C57BL/6 (Wt) recipients			Cxcr1-/- recipients		
**BMT**	**donors**	**C57BL/6 (Wt**	**Cxcr1-/-**	**Cxcr2+/-**	**C57BL/6 (Wt**	**Cxcr1-/-**	**Cxcr2+/-**
		2	6	6	3	6	1
		6	10	0	20	4	1
		10	2	4	16	12	0
		8	6	0	12	22	0
		6	1	1	21	22	0
		1	6	1	6	0	6
		5	7	1	3	11	4
		6	5		5	2	2
		7	0			8	
		11	3			6	
			3				
