# Supplementary material for: NRAS destines tumor cells to the lungs
Source: EMBO Mol Med. 2017 Mar 24;9(5):672–86. doi: 10.15252/emmm.201606978 (PMC5697015; doi:10.15252/emmm.201606978)
Supplement: Supplementary file 9 — Source Data for Figure 8 [file EMMM-9-672-s008.pdf]

Fig 8A **Number of macrometastases of C57BL/6 mice at two weeks after i.v. delivery of 0.25 x 10^6 LLC or AE17 cells preceded and followed by tratment with saline or the CXCR1/2 antagonist navarixin**

LLC

saline	navarixin
9	9
13	8
16	17
17	7
22	4
24	7
13	4
	10
	5

AE17

saline	navarixin
173	135
110	70
120	25
120	20
238	13
178	141
195	82
125	69
	58
	68
