# Supplementary material for: NRAS destines tumor cells to the lungs
Source: EMBO Mol Med. 2017 Mar 24;9(5):672–86. doi: 10.15252/emmm.201606978 (PMC5697015; doi:10.15252/emmm.201606978)
Supplement: Supplementary file 5 — Source Data for Figure 1 [file EMMM-9-672-s004.pdf]

Fig 1E **Number of macrometastases of eleven mouse and human tumor cell lines with known KRAS, NRAS, HRAS, EGFR, BRAF, PIK3CA, TRP53, and STK11 mutation status**

LLC	AE17	MC38	B16F10	PANO2	CT26	AB2	SKMEL2	A549	MDA-MB-23[illegible]	HCC827
10	4	0	0	0	0	0	9	5	0	0
10.5	3	0	0	0	0	0	4	6	0	0
4	3	0	0	0	0	0	13	2	0	0
2.5	20	0	0	0	0	0	3	0	0	0
2	10	0	0	0	0	0	3	2	0	0
9	9	0	0	0	0	0	6	0	0	0
6	13	0	0	0	0	0	4	1	0	0
5	8	0	0	0	0	0	10	2	0	0
7	2	0	0	0	0	0	9	2	0	0
5	5	0	0	0	0	0	9	0	0	0
8	6	0	10	0						
5	2	0	0	0						
3	5	0	0	0						
8	6	0	0	0						
5	4	0	0	0						
7	6	0	0	0						
3		0	0	0						
6		0		0						
4		0								
3		0								
		0								
		0								
		0								
		0								
